# Impact of a Short Educational Session on Early Diagnosis and Management of Acute Kidney Injury for Different Specialties

**DOI:** 10.7759/cureus.57846

**Published:** 2024-04-08

**Authors:** Nikunj Kishore Rout, Subhashree Mishra, Debasis Pathi, Aswini Prasad Patnaik, Sangam Tarun Venkat Mahesh, Eashwar Chand Gundapaneni, Vemula Deepthi

**Affiliations:** 1 Department of Nephrology, Kalinga Institute of Medical Sciences, Bhubaneswar, IND; 2 Department of General Medicine, Kalinga Institute of Medical Sciences, Bhubaneswar, IND

**Keywords:** internal medicine clinical nephrology, medical icu, medical resident education, kidney disease improving global outcomes (kdigo) classification, acute renal injury

## Abstract

Aim and objective: This questionnaire study aimed to evaluate the impact of a short educational session on the early diagnosis and management of acute kidney injury (AKI) among doctors specializing in fields other than nephrology, assessed through pre- and post-test scores. This educational study included resident doctors from various specialties for assessment.

Materials and methods: The study enrolled different specialty resident doctors’ departments and assessed them through questionnaires and assessment scores. The pre-test questionnaires were first distributed and collected after 20 minutes. This was followed by a 30-minute short educational lecture on AKI by the nephrology faculty about its early diagnosis and management as per the Kidney Disease Improving Global Outcomes guidelines. Immediately post continuing medical education, the same questionnaires were distributed along with feedback forms and collected after 10 minutes.

Results: A total of 110 residents participated in the study. All participants showed significant improvement in the post-lecture questionnaires compared to pre-lecture scores. For medicine and allied branch residents, the pre- and post-lecture scores were significantly higher than those of the surgical and allied branch residents. The lowest score was observed in residents of orthopedics. The improvement scores of all departments also showed significant differences. The highest improvement was seen in the department of gynecology, followed by residents of the critical care unit and the department of anesthesia. The residents of those departments with high pre- and post-test scores had lower improvement scores.

Conclusion: The study found a significant knowledge gap in different sister specialties in diagnosing and managing AKI. Short educational sessions showed significant improvement in AKI understanding by addressing the knowledge gaps.

## Introduction

Acute kidney injury (AKI) is an established and common but under-recognized disorder associated with a high risk of mortality, the development of chronic kidney disease (CKD) [1,2] and other organ dysfunction [3]. This clinical syndrome is associated with an abrupt deterioration of kidney function within hours or days, which is often accompanied by oliguria. As per a large cohort study, AKI affects up to 15.3% of all hospitalized patients [4,5]. AKI is associated with a significant increase in in-hospital morbidity, mortality, and costs [6,7]. AKI in tropical and low- and middle-income countries such as India is characterized by a higher burden of community-acquired AKI [8] occurring in relatively young patients without significant comorbidities [2]. Recently published Indian data reveal the spectrum of AKI as post-sepsis (22.5%), post-surgical (21%), post-trauma (28%), post-cardiac events (10.7%), pregnancy-related AKI (15%), and AKI in the pediatric age groups (25.1%) [9,10]. Thus, there is an urgent need to develop health strategies to reduce the enormous and growing burden of AKI and mitigate its outcomes. An Irish government audit showed only 50% of all hospitalized AKI patients had a “good” standard of care. There was inadequate assessment of patients at risk of AKI, and approximately 60% of post-admissions of AKI were predictable, with 21% of AKI being avoidable [11]. Prevention of AKI can be achieved by early detection and treatment as well as adequate follow-up to reduce short-term mortality and the long-term burden of AKI-induced mortality or progression to CKD [6]. The definitions and criteria for AKI have evolved greatly over time, with more than 35 definitions available in a decade [12].

The Acute Dialysis Quality Initiative group developed a system for the diagnosis and classification of a broad range of acute impairments of kidney function through a broad consensus of experts in 2004, popularly called the risk, injury, failure, loss of kidney function, and end-stage kidney disease (RIFLE) criteria [13]. By defining the syndrome of acute changes in renal function more broadly, RIFLE criteria moved beyond established acute renal failure. The term “acute kidney injury/impairment” was proposed to encompass the entire spectrum of the syndrome, from minor changes in markers of renal function to the requirement for renal replacement therapy [14]. AKI not only included acute tubular necrosis but also renal failure and precluding conditions. In 2007, the Acute Kidney Injury Network, an international network of AKI researchers, organized a summit of nephrology and critical care societies from around the world. The group endorsed the RIFLE criteria with a small modification to include small changes in serum creatinine (sCr) (>0.3 mg/dl or >26.5 mmol/l) when they occur within a 48-hour period [14].

According to the Kidney Disease Improving Global Outcomes (KDIGO) definition, AKI is diagnosed by an absolute increase in sCr of at least 0.3 mg/dL (26.5 μmol/L) within 48 hours, a 50% increase in sCr from baseline within seven days, or a urine volume of less than 0.5 mL/kg/h for at least six hours [15]. We wanted to know how much resident doctors in different specialties were conversant with these criteria and find out the utility of a short medical education program in increasing the understanding of the resident doctors about AKI.

In this study, we tried to evaluate the knowledge gaps in the understanding of AKI that hinder its early diagnosis and management, especially in other specialties [16]. As it is well known, AKI may be a part of multi-organ involvement, and every patient with different primary organ involvement admitted under a different specialty cannot be brought under the care of nephrology. Thus, there is an unmet need to address knowledge gaps in the diagnosis of AKI and to increase awareness among the residents of different specialties. This might be a simple means to identify AKI early and thereby lead to the involvement of nephrologists for early management so as to get better outcomes. It is especially important in a developing country like ours, where the doctor-to-patient ratio is low and the nephrologist-to-population ratio is even lower (1.9 per million) [17].

## Materials and methods

Aim and objectives

The study was conducted on the resident doctors of different specialties at the Kalinga Institute of Medical Sciences (KIMS). The primary objective of the study was to assess the impact of short educational sessions on the knowledge of resident doctors. The assessment was done through questionnaires on AKI. The same questionnaire was circulated at the end of the session.

The secondary objectives of the study were to compare the knowledge gaps in understanding AKI between the residents of medicine and allied branches (anesthesia, the critical care unit (CCU), and cardiology) with residents of surgery and allied branches (orthopedics, obstetrics, and gynecology), and further to compare AKI understanding in critical care allied residents (CCU and anesthesia) and residents of non-critical and allied branches (medicine, surgery, orthopedics, obstetrics, gynecology, and cardiology).

Methods

This study was a pre- and post-continuing medical education (CME) questionnaire based on a cross-sectional study. It was conducted by the department of nephrology in seven allied specialties at KIMS, Bhubaneswar.

The study plan and educational session were convened to include as many residents of specialties allied to nephrology as possible, namely, internal medicine, surgery, obstetrics and gynecology, cardiology, anesthesia, CCU, and orthopedics. These seven departments were selected because they most frequently encounter AKI in their patients. Residents who had worked in nephrology within the last 12 months were excluded.

A pre- and post-test assessment was carried out using a validated questionnaire. Three nephrologists drew up the questionnaire that was validated with the opinions of five residents and five other nephrologists from different parts of the country. The residents looked at the ease of understanding the questionnaire (i.e., the face validity), while the experts examined the construct and content validity. The participants were first seated in a seminar hall, and the pre-CME questionnaire was distributed and had to be answered within 20 minutes. This was followed by a 30-minute structured teaching session on AKI by a nephrology faculty member. The questionnaire was the same for all the study participants. The CME covered the basic definition, classification, and understanding of AKI and its management, with a particular focus on the AKI scenarios faced by the particular departments. This was followed by a post-lecture questionnaire, and feedback was taken from the participants.

Sample size

A convenient sample was taken, and the sample’s power was calculated post hoc. Data were analyzed using SPSS Statistics version 24.0 (IBM Corp. Released 2016. IBM SPSS Statistics for Windows, Version 24.0. Armonk, NY: IBM Corp.). The continuous variables were subjected to the Shapiro-Wilk test for normality. The normally distributed variables were subjected to parametric tests like paired sample “t” tests, independent sample “t” tests, and ANOVA for comparison of means. Non-parametric tests used included the Wilcoxon signed rank and Mann-Whitney U test for comparison of continuous variables. The cut-off value of p<0.05 was considered significant in all tests.

## Results

Table 1 presents a pairwise comparison of pre- and post-lecture scores. The mean ± SD and median (IQR) of the post-lecture score (76.0 ± 11.6, 77.8 (70.4-83.8)) were significantly higher than the pre-lecture mean ± SD and median (IQR) score (51.4 ± 16.2, 53.7 (40.7 ± 63.0)) with p=0.000. The mean and median improvement in the post-lecture score were 24.3 ± 14.5 and 22.2 (14.8-31.5), respectively, with a range from 0.0 to 75.9.

**Table 1:** Pairwise comparison of different time points (N=110) * Wilcoxon signed ranks test "p" value

Time point	Knowledge score in percentage		
	Mean ± SD	Median (IQR)	Range
Pre-lecture	51.4 ± 16.2	53.7 (40.7-63.0)	(9.3-83.3)
Post-lecture	76.0 ± 11.6	77.8 (70.4-83.8)	(33.3-98.2)
Improvement	24.3 ± 14.5	22.2 (14.8-31.5)	(0.0-75.9)
Pre- vs. post-lecture	0.000*		

Among the surgical and allied branches (orthopedics, obstetrics, and gynecology), the mean ± SD and median (IQR) of the pre-lecture score (45.8 ± 14.9, 48.2 (38.9-57.4)) were significantly lower than the post-lecture score (71.7 ± 12.5, 74.1 (66.7-81.5)), with p=0.000. Similarly, in the medicine and allied branches (anesthesia, CCU, and cardiology), the mean ± SD and median (IQR) at the pre-lecture score (56.3 ± 15.7, 61.1 (42.6-66.7)) were significantly lower than the post-lecture score (79.7 ± 9.3, 81.5 (75.9-85.2)), with p=0.000.

In the medicine and allied branches, the median (IQR) of pre- and post-lecture was significantly higher than in the surgical and allied branches (p<0.05). Both the surgical and allied and medicine and allied branches witnessed improvement in the post-lecture score. The improvement did not have a significant difference between the two groups (p=0.325). Table 2 depicts the details.

**Table 2:** Comparison by group and comparison between pre- and post-test * Wilcoxon signed ranks test "p" value

Time point	Knowledge score in percentage						Mann–Whitney U “p” value
	Medicine						
	Surgical and allied (N=51)			Medicine and allied (N=59)			
	Mean ± SD	Median (IQR)	Range	Mean ± SD	Median (IQR)	Range	
Pre-lecture	45.8 ± 14.9	48.2 (38.9-57.4)	(9.3-70.4)	56.3 ± 15.7	61.1 (42.6-66.7)	(18.5-83.3)	0.000
Post-lecture	71.7 ± 12.5	74.1 (66.7-81.5)	(33.3-87.0)	79.7 ± 9.3	81.5 (75.9-85.2)	(53.7-98.2)	0.001
Improvement	26.0 ± 16.6	24.1 (14.8-33.3)	(0.0-75.9)	22.9 ± 12.3	18.5 (14.8-31.5)	(1.9-55.6)	0.325
Pre- vs. post-lecture	0.000*			0.000*			

In non-CCU residents, the mean ± SD of the post-lecture score was 76.0 ± 12.3, which was significantly higher than the pre-lecture score (52.8 ± 16.5), with p=0.000. Similarly, in CCU residents, the mean ± SD of the post-lecture score was 76.0 ± 9.5, significantly higher than the pre-lecture score (48.0 ± 14.8), with p=0.000.

The mean ± SD at pre-lecture was 52.8 ± 16.5 in non-ICU residents and 48.0 ± 14.8 in ICU residents, and the difference was not significant (p=0.160). The post-lecture mean ± SD was 76.0 ± 12.3 in non-CCU residents and 76.0 ± 9.5 in CCU residents, and the difference was not significant (p=0.989). The improvement in post-lecture scores was very high in both groups. The improvement mean ± SD was 22.9 ± 14.6 in non-CCU residents and 28.0 ± 13.5 in CCU residents, and the difference was not significant (p=0.095). Table 3 presents the details.

**Table 3:** Comparison of scores between CCU and non-CCU residents * Paired sample "t" test "p" value CCU: critical care unit

Time point	Knowledge score in percentage						Independent sample test “p” value
	CCU						
	Non-CCU residents			CCU residents			
	N	Mean	SD	N	Mean	SD	
Pre-lecture	79	52.8	16.5	31	48.0	14.8	0.160
Post-lecture	79	76.0	12.3	31	76.0	9.5	0.989
Improvement	79	22.9	14.6	31	28.0	13.5	0.095
Pre- vs. post-lecture	0.000*			0.000*			

Table 4 presents a comparison of pre- and post-lecture scores among departments. During pre-lecture, the overall mean ± SD score was 51.4 ± 16.2. The scores of different departments were significantly different (p=0.000). The highest pre-lecture score was observed for the residents of the department of medicine (67.4 ± 8.5), followed by the department of cardiology (63.9 ± 11.7) and the department of anesthesia (52.0 ± 15.2). The lowest score was observed among the residents of CCU (43.2 ± 13.7). During the post-lecture, the overall mean ± SD score was 76.0 ± 11.6. The scores of different departments were significantly different (p=0.000). The highest post-lecture score was observed for the residents of the department of medicine (84.7 ± 8.7), followed by cardiology (81.9 ± 5.6) and anesthesia (79.4 ± 7.7). The lowest score was observed among the residents of the department of orthopedics (68.7 ± 14.2).

**Table 4:** Comparison of pre- and post-lecture scores among departments CCU: critical care unit, ANOVA: analysis of variance

Time point	Department	Knowledge score in percentage			ANOVA “p” value
		N	Mean	SD	
Pre-lecture	Surgery	22	45.5	11.3	0.000
	Gynecology	17	43.9	17.6	
	Medicine	18	67.4	8.5	
	CCU	15	43.2	13.7	
	Anesthesia	15	52.0	15.2	
	Cardiology	10	63.9	11.7	
	Orthopedics	13	48.6	17.2	
	Total	110	51.4	16.2	
Post-lecture	Surgery	22	69.7	13.8	0.000
	Gynecology	17	77.8	6.1	
	Medicine	18	84.7	8.7	
	CCU	15	72.0	10.0	
	Anesthesia	15	79.4	7.7	
	Cardiology	10	81.9	5.6	
	Orthopedics	13	68.7	14.2	
	Total	110	76.0	11.6	
Improvement	Surgery	22	23.4	10.7	0.002
	Gynecology	17	34.6	20.9	
	Medicine	18	17.7	7.4	
	CCU	15	28.8	15.1	
	Anesthesia	15	27.4	12.8	
	Cardiology	10	16.7	7.9	
	Orthopedics	13	18.9	14.1	
	Total	110	24.3	14.5	

The overall mean ± SD score of improvement was 24.3 ± 14.5. The improvement scores of different departments were significantly different (p=0.002). The highest improvement score was observed for the residents of the department of gynecology (34.6 ± 20.9), followed by the CCU (28.8 ± 15.1) and the department of anesthesia (27.4 ± 12.8). The lowest improvement score was observed among the residents of the department of cardiology (16.7 ± 7.9). The residents of those departments with high pre- and post-lecture scores had lower improvement, and those with low levels of improvement scores had low mean improvement scores.

On analysis of all residents as per the questions asked (Table 5), we had some more understanding of the areas of AKI that needed more sensitization. Around 50% of all participants failed to define any one of the three criteria of AKI as per the KDIGO definition. In most of the departments, particularly the ICU and department of surgery, all but a quarter of participants were able to categorize AKI into pre-, intra-, and post-renal categories. Most participants were able to enumerate the causes of community- and hospital-acquired AKI. The surgery, gynecology, and orthopedics residents, who were not able to enumerate two out of five of these causes in the pre-lecture questionnaire, showed a marked improvement post-lecture. Most departments’ residents were able to enumerate the common nephrotoxic drugs and the risk factors for AKI (e.g., old age, hypotension, dehydration, use of nephrotoxic agents, and the presence of CKD). However, most failed to enumerate the clinical signs that they needed to see in cases of suspected AKI.

**Table 5:** Comparison of questionnaire item scores between pre- and post-test by respective departments AKI: acute kidney injury, OPD: outpatient department, ICU: intensive care unit

Questionnaire item	Department													
	Surgery (N=22)		Gynecology (N=17)		Medicine (N=18)		ICU (N=15)		Anesthesia (N=15)		Cardiology (N=10)		Orthopedics (N=13)	
	Pre	Post	Pre	Post	Pre	Post	Pre	Post	Pre	Post	Pre	Post	Pre	Post
	Mean ± SD	Mean ± SD	Mean ± SD	Mean ± SD	Mean ± SD	Mean ± SD	Mean ± SD	Mean ± SD	Mean ± SD	Mean ± SD	Mean ± SD	Mean ± SD	Mean ± SD	Mean ± SD
What is the definition of AKI?	0.55 ± 0.74	2.23 ± 1.02	0.29 ± 0.59	2.71 ± 0.47	2.17 ± 1.20	2.78 ± 0.55	0.60 ± 0.74	2.00 ± 1.20	0.80 ± 0.86	2.67 ± 0.49	0.90 ± 0.88	2.90 ± 0.32	0.54 ± 0.97	2.23 ± 1.09
What are the types of AKI?	1.09 ± 1.41	3.00 ± 0.00	2.00 ± 1.41	2.82 ± 0.73	3.00 ± 0.00	3.00 ± 0.00	1.67 ± 1.18	2.33 ± 1.23	3.00 ± 0.00	3.00 ± 0.00	1.90 ± 1.45	3.00 ± 0.00	2.23 ± 1.30	2.77 ± 0.83
What are the common causes of community-acquired AKI?	0.68 ± 0.65	3.64 ± 1.22	1.71 ± 1.76	4.82 ± 0.39	3.39 ± 1.20	4.78 ± 0.55	1.27 ± 1.03	3.53 ± 1.64	1.67 ± 1.54	3.87 ± 1.36	2.80 ± 1.99	4.20 ± 0.92	1.69 ± 1.60	2.38 ± 1.98
What are the common causes of hospital-acquired AKI?	1.32 ± 1.17	2.86 ± 1.32	1.65 ± 1.12	4.18 ± 1.02	2.89 ± 0.83	4.78 ± 0.55	1.33 ± 1.11	3.40 ± 1.50	2.53 ± 0.92	4.00 ± 0.85	3.20 ± 1.14	4.60 ± 0.70	2.08 ± 1.50	3.62 ± 1.90
Name any three nephrotoxic drugs.	1.86 ± 1.08	2.77 ± 0.53	2.18 ± 0.88	3.00 ± 0.00	2.89 ± 0.32	3.00 ± 0.00	1.27 ± 1.03	3.00 ± 0.00	2.33 ± 0.82	3.00 ± 0.00	2.70 ± 0.68	3.00 ± 0.00	2.00 ± 1.00	2.69 ± 0.86
Name five major risk factors for AKI.	2.00 ± 1.51	2.68 ± 1.62	1.59 ± 1.06	4.00 ± 1.06	2.17 ± 1.38	4.44 ± 0.62	1.20 ± 1.01	3.20 ± 2.04	2.73 ± 1.16	4.27 ± 1.03	3.70 ± 0.95	4.40 ± 0.70	1.69 ± 1.49	3.54 ± 1.85
Name five clinical findings to be seen in a case suspected of AKI.	0.91 ± 0.68	1.27 ± 0.46	1.00 ± 0.61	1.65 ± 0.49	1.33 ± 0.49	1.83 ± 0.38	0.60 ± 0.74	1.27 ± 0.70	1.13 ± 0.52	1.47 ± 0.52	1.10 ± 0.32	1.20 ± 0.79	0.85 ± 0.56	1.15 ± 0.69
What things should be monitored in the case of AKI?	1.45 ± 1.82	2.18 ± 2.04	1.12 ± 1.69	2.35 ± 2.03	2.67 ± 1.94	3.33 ± 1.53	0.87 ± 1.64	1.87 ± 2.07	0.80 ± 1.66	2.93 ± 1.83	1.20 ± 1.93	3.20 ± 1.69	1.85 ± 2.08	2.46 ± 2.03
What investigations should be advised in a case of AKI?	2.18 ± 1.40	3.00 ± 1.11	1.94 ± 1.03	1.82 ± 1.07	2.22 ± 0.94	2.61 ± 1.20	1.40 ± 1.35	2.00 ± 1.13	2.27 ± 1.22	2.40 ± 1.06	3.00 ± 0.82	2.70 ± 1.06	2.46 ± 0.88	2.15 ± 1.46
How would you manage a pre-renal AKI?	2.14 ± 1.04	2.55 ± 0.74	1.65 ± 1.12	2.71 ± 0.85	3.06 ± 0.80	3.17 ± 0.86	1.47 ± 1.25	2.80 ± 1.21	2.00 ± 1.07	2.33 ± 0.72	2.70 ± 1.16	2.70 ± 0.48	2.46 ± 1.45	2.62 ± 0.77
What are the indications for renal replacement therapy in AKI?	2.91 ± 1.48	3.36 ± 1.22	2.71 ± 1.11	3.47 ± 0.72	3.56 ± 0.62	3.56 ± 0.71	2.07 ± 1.44	3.07 ± 1.22	2.93 ± 1.10	3.73 ± 0.46	3.90 ± 0.32	3.80 ± 0.42	3.31 ± 1.18	3.38 ± 0.96
What measures should be taken to prevent AKI in the hospital (OPD/indoor)?	2.95 ± 1.36	2.95 ± 1.40	3.18 ± 1.63	3.41 ± 0.80	3.72 ± 1.07	3.22 ± 1.11	1.33 ± 1.18	2.73 ± 1.34	3.27 ± 1.16	3.80 ± 0.56	4.30 ± 1.06	4.10 ± 0.57	3.00 ± 1.58	3.08 ± 1.19
What measures should be taken for the prevention of contrast-induced AKI?	1.64 ± 1.14	2.68 ± 1.17	1.82 ± 1.02	3.76 ± 0.56	1.61 ± 1.04	3.83 ± 0.38	1.67 ± 1.40	3.07 ± 1.22	1.60 ± 0.83	3.93 ± 0.26	1.80 ± 1.14	3.90 ± 0.32	1.62 ± 1.12	3.77 ± 0.44
What nutritional advice should be given to AKI patients who are not on dialysis?	1.05 ± 1.00	1.64 ± 1.14	1.06 ± 1.14	1.18 ± 0.73	1.89 ± 0.90	1.33 ± 1.03	1.07 ± 0.96	1.27 ± 0.80	1.20 ± 0.78	1.40 ± 0.63	1.60 ± 1.08	1.50 ± 0.71	0.62 ± 0.87	0.85 ± 0.99

Most residents were aware of the investigations that help to diagnose AKI (e.g., renal function tests, routine urine examination, renal ultrasonography, and management of pre-renal AKI through volume replacement by crystalloids, colloids, blood products, and inotropes). Most were unable to list the measures to reduce AKI in hospital/clinical settings (e.g., volume status assessment, measurement of vitals, urine output, management of sepsis, and avoiding nephrotoxic agents). Similarly, there was less pre-lecture response regarding the prevention of contrast-induced AKI (e.g., risk stratification of the patient, use of iso-osmolar contrast, pre-procedure hydration, minimal use of contrast, and the like, which is one of the most common causes of hospital-acquired AKI). Most residents were able to enumerate more than two causes of renal replacement therapy requirements (e.g., uremic features, severe hyperkalemia, metabolic acidosis, and fluid overload not responding to medical management).

## Discussion

In the last 10 years, much emphasis has been put on the early detection and treatment of AKI. The International Society of Nephrology has come up with an ambitious “no preventable AKI by 2025,” which is the 0 by 25 Initiative [18]. In this endeavor to raise awareness of AKI, it was decided to conduct a CME on AKI diagnosis and management to address the knowledge gaps among residents of different specialties other than nephrology. A pre- and post-lecture questionnaire was used to measure the impact of this study. Through comparative analysis of scores in different surgical and medical specialties and in ICU residents and non-ICU residents, the goal was to find out if there was any difference in the level of awareness in residents of different specialties as assessed through pre-lecture scores. The post-lecture score comparisons pointed toward the impact of the lecture, and the improvement scores were also estimated and compared to assess differences in residents’ short-term grasping ability.

On analyzing the pre- and post-questionnaires (Table 1), it was found that there was a significant improvement (p<0.000) in the understanding of AKI after the short educational session. The mean pre-lecture score of all residents improved from 51.4% ± 16.2 to 76.0% ± 11.6, showing an improvement of 24.3% ± 14.5. Hence, the primary objective to assess the impact of the study showed a significant improvement in the understanding of AKI. A significant improvement was maintained (p=0.000) even when we assessed medicine and allied specialties separately from surgical and allied specialties.

On comparing the mean pre- and post-lecture scores among residents from the medicine and allied branches versus those from the surgery and allied branches (Table 2), the difference was significant (p<0.05). This showed a better understanding of AKI by medicine and allied branch residents than by surgical and allied branch residents. Both the baseline and post-session understanding of AKI were better in medicine and allied branches than in surgical and allied branches.

The mean pre- and post-lecture score between ICU versus non-ICU residents when compared was not significant (p<0.05). These results were contrary to expectations, as ICU residents are usually trained to diagnose and manage AKI early. The post-lecture scores were also non-significant. This showed the need for more sensitization classes for ICU residents regarding AKI diagnosis and management.

Table 4 shows a comparison of pre- and post-lecture scores among departments. Pre-lecture, the overall mean ± SD score was 51.4 ± 16.2. The scores of different departments were significantly different (p=0.000). This indicated a significant difference in baseline knowledge of AKI understanding among residents of participating departments. The high pre-lecture-scoring departments such as medicine (67.4 ± 8.5), cardiology (63.9 ± 11.7), and anesthesia (52.0 ± 15.2) had better baseline knowledge than the others, and the lowest-scoring residents were from the ICU (43.2 ± 13.7). Post-lecture, the overall mean ± SD score was 76.0 ± 11.6, and the post-lecture scores of different departments also differed significantly (p=0.000). The highest post-lecture score, suggesting the biggest impact, was received by the residents of the department of medicine (84.7 ± 8.7), followed by the department of cardiology (81.9 ± 5.6) and the department of anesthesia (79.4 ± 7.7). The lowest score was observed among the orthopedic residents (68.7 ± 14.2). These comparative observations not only showed the difference in baseline knowledge of AKI among the residents of different departments but also helped to target departments that needed frequent sensitization. This served as an audit for AKI awareness and understanding, diagnosis, and management.

Upon assessing the improvement in knowledge or awareness of AKI depicted by the overall improvement score, ± SD was 24.3 ± 14.5. The improvement scores of different departments were significantly different (p=0.002). The highest improvement score was observed for the gynecology residents (34.6 ± 20.9), followed by those from the ICU (28.8 ± 15.1) and anesthesia (27.4 ± 12.8). The lowest improvement score was observed among the cardiology residents (16.7 ± 7.9). The residents of those departments with high pre- and post-lecture scores had lower improvement scores, and those with low levels of improvement scores had low mean improvement scores.

The analysis of residents' understanding of AKI provides valuable insights into areas that require attention and improvement within medical education and clinical practice. These study findings prompt a discussion on the importance of continuous education, targeted sensitization efforts, and the implications for patient care and outcomes.

One of the key observations from the analysis is the significant proportion of residents (Table 5), particularly those outside nephrology specialties, who struggled to define the diagnostic criteria for AKI according to the KDIGO guidelines [1]. This highlights a fundamental gap in knowledge that could potentially lead to delayed diagnosis and management of AKI, a condition associated with high morbidity and mortality rates. It underscores the critical need for repeated sensitization efforts to ensure all healthcare professionals, regardless of specialty, possess a comprehensive understanding of AKI criteria.

Furthermore, while residents generally demonstrated an understanding of categorizing AKI and enumerating its causes, there were notable discrepancies among different departments. For instance, residents in departments like surgery, gynecology, and orthopedics showed marked improvement post-lecture in enumerating causes of AKI. This suggests that targeted educational interventions can effectively address knowledge gaps in specific clinical contexts.

Another concerning finding is the lack of awareness among residents regarding clinical signs indicative of suspected AKI. Early recognition of AKI symptoms is crucial for prompt intervention and preventing disease progression. Therefore, efforts to enhance residents' ability to recognize and interpret clinical signs associated with AKI should be prioritized.

Moreover, while residents demonstrated awareness of diagnostic investigations for AKI, such as renal function tests and urine examinations, there was a notable deficiency in understanding measures to reduce AKI risk in hospital settings and prevent contrast-induced AKI. Given the preventable nature of some AKI cases, comprehensive education on preventive strategies is imperative to improve patient outcomes and reduce healthcare-associated complications.

The study's findings also underscore the importance of interdisciplinary collaboration in AKI management. AKI often requires a multidisciplinary approach involving nephrologists, intensivists, surgeons, and other healthcare professionals. Effective communication and shared understanding of AKI among different specialties are essential for coordinated care delivery and optimal patient outcomes.

We also reviewed the feedback given by the participating residents, which showed that 87% of residents had attended a CME workshop on AKI for the first time. This study did show that basic Bachelor of Medicine and Bachelor of Surgery and even post-graduate courses have little to offer residents in the early diagnosis of AKI and its management, and there is a need to sensitize the other specialties toward AKI. This will not only help surgeons develop good post-operative recovery plans for patients but will also save the financial burden arising due to renal failure management and prolonged hospital stays.

Of such seminars, 70% of participants were believed to be helpful in addressing the deficiencies in knowledge regarding AKI, and these should be cyclically done to train sister departments. Nearly 100% of participants were satisfied with the content of the presentation. Approximately two-thirds of them believed the CME to be helpful in sensitization.

The limitation of the study stems from it being conducted at a single institute, involving a relatively small sample size, and based on a brief educational session.

## Conclusions

This study had relevance in spreading awareness to stop AKI and preventable AKI. The surgery and allied branches (obstetrics, gynecology, and orthopedics) need to be sensitized more often regarding the early diagnosis, treatment, and early referral of AKI. This can be done through seminars, short ICU training, short nephrology training, and so on. The ICU is supposed to detect AKI early and take preventive measures; however, the ICU appears to have comparatively low baseline scores in the individual department analysis. The pre-lecture score was lowest in ICU residents, which points to the need for more intensive training regarding early AKI diagnosis and quick management of AKI.

There is a need for repeated short educational sessions, CMEs/lectures, especially in different departments, to spread awareness of AKI. This study suggests that the positive impact of this training will help to attain the 0 by 25 Initiative of the International Society of Nephrology to eliminate all preventable deaths from AKI by 2025.

## Appendices

Utility of a short educational session on acute kidney injury among resident doctors of different specialties

Questionnaire

Designation:

Department:

1. Definition of AKI.

a. Increase in serum creatinine more than ………………… mg/dl within 48 hours

b. Increase in serum creatinine more than ………………… times the baseline which is presumed to have occurred in the past seven days.

c. Decrease in urine output less than …………… ml/kg/hr for six hours.

2. Types of AKI.

a.      …………………………………

b.      …………………………………

c.       ………………………………….

3. Common causes of community-acquired AKI (any 5).

a.      …………………………………….

b.      ……………………………………..

c.       ……………………………………….

d.      …………………………………………

e.      ………………………………………..

4. Common causes of hospital-acquired AKI (any 5).

a.      ………………………………………….

b.      …………………………………………….

c.       ……………………………………………..

d.      ……………………………………………..

e.      ………………………………………………

5. Name any three nephrotoxic drugs.

a.      ………………………………………………

b.      ………………………………………………..

c.       ………………………………………………..

6. Name five major risk factors for AKI.

a.      ………………………………………………

b.      ………………………………………………

c.       ………………………………………………..

d.      ………………………………………………..

e.      ………………………………………………..

7. Name five clinical findings to be seen in a case suspected of AKI.

a.      ………………………………………………..

b.      ……………………………………………….

c.       ………………………………………………..

d.      …………………………………………………

e.      …………………………………………………

8. Things to be monitored in the case of AKI.

a.      ………………………………………………….

b.      ………………………………………………….

c.       ………………………………………………….

9. Investigations to be advised in a case of AKI.

a.      ……………………………………………………

b.      …………………………………………………..

c.       ………………………………………………

d.      ………………………………………………

10. How will you manage a prerenal AKI?

a.      ………………………………………………

b.      …………………………………………………

c.       …………………………………………………..`

d.      …………………………………………………

11. Indications for renal replacement therapy in AKI.

a.      ………………………………………………….

b.      ………………………………………………….

c.       …………………………………………………….

d.      …………………………………………………….

e.      …………………………………………………….

12. Measures to be taken to prevent AKI in the hospital (OPD/indoor).

a.      ………………………………………………….

b.      …………………………………………………

c.       …………………………………………………….

d.      ………………………………………………………….

13. Measures to be taken for prevention of contrast-induced AKI.

a.      ………………………………………………….

b.      …………………………………………………..

c.       …………………………………………………

d.      …………………………………………………..

14. Nutritional advice for AKI patients who are not on dialysis?

a.      Calorie intake………………..

b.      Protein intake………………………

c.      Salt intake………………………..

d.      Fluid intake……………………….
